# Southern Students Leaving the Medical Schools of Philadelphia

**Published:** 1860-01

**Authors:** 


### SOUTHERN STUDENTS LEAVING THE MEDICAL SCHOOLS OF PHILADELPHIA.

We have learned through the newspapers that over three hundred medical students from the southern states, recently left Philadelphia in a body for Richmond, and other southern cities.

If this movement is to be regarded as the result of political causes, it is to be regretted. If, on the contrary, it was the result of a returning sense of the mistake the young gentlemen committed in passing by the schools of their own states, especially for those of Pennsylvania, we cannot but commend their good judgment.

The Medical Schools of the southern cities suffer nothing in comparison with any in this or foreign countries.

Their professors are learned and eloquent; the facilities for clinical teaching, are in some of the large cities, ample. Why then, should they not be supported by southern students?

We do not in any way intend to disparage the advantages of Philadelphia as a seat of medical learning. Her character is too well established to require endorsement or fear depreciation, but we have long believed that a more equal distribution of students among such medical schools of the country as possess facilities for clinical instruction instead of gathering five hundred on the seats of a single lecture room, would be for the advantage of all concerned, and we hope that hereafter the medical students of the West will not be behind those of the South in their attachment to the institutions of their own particular sections; institutions which, if they are not already fully equal to any others, only require the patronage to which they are legitimately entitled to render them so.

